# Quantitative monitoring of circulating tumor DNA predicts response of cutaneous metastatic melanoma to anti-PD1 immunotherapy

**DOI:** 10.18632/oncotarget.25404

**Published:** 2018-05-18

**Authors:** Guillaume Herbreteau, Audrey Vallée, Anne-Chantal Knol, Sandrine Théoleyre, Gaelle Quéreux, Emilie Varey, Amir Khammari, Brigitte Dréno, Marc G. Denis

**Affiliations:** ^1^ Laboratoire de Biochimie et Plateforme de Génétique Moléculaire des Cancers, CHU Nantes, Nantes, France; ^2^ Centre de Recherche en Cancérologie et Immunologie, CRCINA, INSERM U1232, Nantes, France; ^3^ Service de Dermatologie, CHU Nantes, Nantes, France; ^4^ Centre d’Investigation Clinique INSERM CIC1413, CHU Nantes, Nantes, France

**Keywords:** metastatic melanoma, anti-PD1, circulating tumor DNA, cell-free DNA, digital PCR

## Abstract

Immunotherapies have changed the medical management of metastatic melanoma. However, the early detection of patients who do not respond to these treatments is a key issue. We evaluated the quantitative monitoring of circulating tumor DNA (ctDNA) as an early predictor of response to anti-PD1. Patients treated with anti-PD1 for metastatic mutated melanoma were selected. The somatic alteration detected on the tumor tissue was quantified on plasma DNA by digital PCR (dPCR) at treatment initiation, after 2 and 4 weeks of treatment, and then every 4 weeks until progression. The absence of biological response (defined as a significant decrease in the amount of ctDNA relative to the baseline level) after 2 weeks of treatment was associated with a lack of clinical benefit under anti-PD1. In the presence of a biological response at week 2, detection of subsequent biological progression (significant increase in the amount of ctDNA relative to its nadir) was 100% predictive of progressive disease, on average 75 days prior to radiological detection. Patients with a persistent biological response beyond week 16 did not experience any progressive disease and exhibited sustained responses. In conclusion, we show that quantitative monitoring of ctDNA, using criteria accounting for dPCR measurement imprecision, allows the early and specific detection of patients who do not respond to anti-PD1 therapy.

## INTRODUCTION

Immunotherapies have changed the medical management of metastatic cutaneous melanoma fundamentally. Anti-Programmed-Death receptor 1 (PD1) antibodies, alone or in combination with anti-CTLA-4 antibodies have increased both progression free survival (PFS) and overall survival (OS) markedly [1–5]. Nevertheless, 56 to 73% of patients do not respond to anti-PD1 [1–3], and early identification of these patients remains one of the major challenges associated with such treatments. None of the markers evaluated so far have demonstrated a sufficient predictive value to be able to guide the therapeutic strategy [6–9]. In particular, PD-L1 tumor expression cannot exclude a possible response to anti-PD1 with response rates of 43 to 58% in PD-L1 positive patients, and 13 to 41% in PD-L1 negative patients [1–3, 10, 11].

In this context, the identification of non-responders to anti-PD1 is, at the present time, solely based on radiological and clinical monitoring. However, some patients can experience pseudo progression which might be difficult to differentiate from true progression and delay the detection of primary resistance to anti-PD1 [12, 13].

These issues have aroused strong interest in the development of tumor markers specific for metastatic melanoma, and in particular circulating tumor DNA (ctDNA). Indeed, ctDNA is quantitatively correlated with the baseline tumor burden, and several studies have shown the existence of a correlation between the variations of the ctDNA level and clinical progression during follow-up [14–17].

The use of ctDNA to monitor the response of melanoma to anti-PD1 immunotherapy requires the development of criteria for interpreting longitudinal variations in ctDNA. Recently, Lee *et al.* proposed qualitative criteria based on the detectability of ctDNA: the persistence of detectable ctDNA up to the 12th week of treatment was associated with a response rate of 6%, compared to 74% for patients whose ctDNA was initially undetectable or became undetectable before week 12 [17]. Nevertheless, the imperfect discrimination of non-responders and the 12-week delay required to identify them, limits the clinical relevance of these qualitative criteria.

The aims of our study were to establish evaluation criteria of ctDNA variations, and to determine whether ctDNA monitoring could enable reliable, early detection of the response to anti-PD1 of patients with metastatic cutaneous melanoma.

## RESULTS

### Patients

This study included 53 patients with stage IV or non-resectable stage IIIc metastatic cutaneous melanoma with a *BRAF* or a *NRAS* mutation. The patients’ baseline characteristics are presented in Table 1. Forty-nine patients were treated with nivolumab monotherapy and 4 patients were treated with a combination of nivolumab and ipilimumab. A *BRAF* codon 600 mutation was found in 24 patients: 2 were treated with nivolumab as a first-line treatment, 17 had been previously treated with BRAF inhibitor, and 5 received a BRAF inhibitor and then ipilimumab prior to nivolumab. Twenty-nine patients had an *NRAS* mutation: 24 received nivolumab as a first-line treatment, 2 were previously treated with ipilimumab, and 3 received ipilimumab and chemotherapy. The nature of the treatment preceding nivolumab is summarized in Table 1. The median follow-up period was 6.8 months (range = 3.7–25.7 months).

**Table 1:** Patients baseline characteristics

	Total (*n* = 53)	OR+Group (*n* = 19)	OR-PD+Group (*n* = 20)	OR-PD−Group (*n* = 14)	
Age (years)					
Median (range)	64 (27–90)	65 (35–82)	64 (31–90)	61,5 (27–82)	*P* = 0.837
Sex					
Female	24 (45.3%)	11 (57.9%)	7 (35.0%)	6 (42.9%)	*P* = 0.204
Male	29 (54.7%)	8 (42.1%)	13 (65.0%)	8 (57.1%)	
Stage					
III non-resectable	11 (20.8%)	4 (21.1%)	3 (15.0%)	4 (28.6%)	*P* = 0.793
IV	42 (79.2%)	15 (78.9%)	17 (85.0%)	10 (71.4%)	
Somatic mutation					
*BRAF* (codon 600)	24 (45.3%)	7 (36.8%)	9 (45.0%)	8 (57.1%)	*P* = 0.621
*NRAS* (exon 2 or 3)	29 (54.7%)	12 (63.2%)	11 (55.0%)	6 (42.8%)	
Tumor thickness (mm)					
Mean ± SD	3.04 ± 2.20	2.78 ± 1.25	3.23 ± 2.83	3.08 ± 2.25	*P* = 0.971
Ulceration					
No	21 (39.6%)	6 (31.6%)	10 (50.0%)	5 (35.7%)	*P* = 0.346
Yes	15 (28.3%)	5 (26.3%)	4 (20.0%)	6 (42.9%)	
Unknown	17 (32.1%)	8 (42.1%)	6 (30.0%)	3 (21.4%)	
Treatment					
Nivolumab	49 (92.5%)	17 (89.5%)	19 (95.0%)	13 (92.9%)	*P* = 0.788
Nivo + Ipilimumab	4 (7.5%)	2 (10.5%)	1 (5.0%)	1 (7.1%)	
Previous treatment					
None (1st line)	26 (49.1%)	11 (57.9%)	9 (45.0%)	6 (42.9%)	*P* = 0.664
Chemotherapy	2 (3.8%)	1 (5.3%)	1 (5.0%)	0	
Immunotherapy	5 (9.4%)	3 (15.8%)	1 (5.0%)	1 (7.1%)	
Targeted therapy	20 (37.7%)	4 (21.0%)	9 (45.0%)	7 (50.0%)	
Number of metastatic sites					
Mean ± SD	4.7 ± 3.3	4.4 ± 3.3	5.3 ± 3.4	4.2 ± 3.4	*P* = 0.340
Baseline LDH (UI/L)					
Mean ± SD	246 ± 222	185 ± 66	255 ± 149	314 ± 385	*P* = 0.319
ctDNA detectability					
Detectable ctDNA	23 (43.4%)	6 (31.6%)	11 (55.0%)	6 (42.8%)	*P* = 0.337
Undetectable ctDNA	30 (56.6%)	13 (70.4%)	9 (45.0%)	8 (57.2%)	
ctDNA concentration (mutated copies/mL)					
Mean ± SD	857 ± 3695	91 ± 195	581 ± 1079	2291 ± 7047	*P* = 0.330

Thirty-one patients (58%) were alive at the time of analysis, and 21 patients (40%) had an ongoing treatment response. For the total cohort, the response rate (RR) was 36%, the PFS at 1 year was 46%, and the OS at 1 year was 63%.

A total of 262 samples were analyzed, including a baseline sample for all 53 patients, collected prior to anti-PD1 initiation. Among these 53 patients, 19 patients presented an objective response (OR) as per RECIST during treatment (10 CR and 9 PR), without presenting any tumor progression at the time of analysis (OR+ group, Table 1). Twenty patients had progressive disease (PD) without any objective response (OR-PD+ group, Table 1). Finally, 14 patients had left the study before an objective response or a progressive disease could be observed (OR–PD− group, Table 1): 7 died before the first evaluation, 2 patients experienced grade 4 adverse effects (acute kidney transplant rejection with autoimmune hemolytic anemia, and pancytopenia), 1 patient was lost to follow-up, and 4 patients showed persistent tumor stability until the time of analysis.

The clinical characteristics of these three groups were similar in terms of age, gender, stage of disease, nature of mutation, primary tumor thickness and ulceration, nature of treatment, number and nature of previous treatments, number of metastatic sites and the baseline LDH activity (Table 1).

### Baseline ctDNA

ctDNA was detectable at baseline in 23/53 (43%) patients, including 6/19 (31.6%) OR+ patients, 11/20 (55%) OR–PD+ patients and 6/14 (42.8%) OR-PD− patients. The ctDNA detectability did not differ significantly between these 3 groups (*P* = 0.337). None of the patient characteristics were associated with ctDNA detectability (Table 2).

**Table 2:** Association of baseline ctDNA detectability with clinicopathological features

	Detectable cthNA (*n* = 23)	Undetectable cthNA (*n* = 30)	
Age (years)			
Median (range)	71 (27–90)	61.5 (31–85)	*P* = 0.389
Sex			
Female	7 (30.4%)	17 (56.7%)	*P* = 0.057
Male	16 (69,6)	13 (43,3)	
Stage			
III non-resectable	3 (13.0%)	8 (26.7%)	*P* = 0.225
IV	20 (87.0%)	22 (73.3%)	
Somatic mutation			
BRAF (codon 600)	11 (47.8%)	13 (43.3%)	*P* = 0.745
NRAS (exon 2 or 3)	12 (52.2%)	17 (56.7%)	
Tumor thickness (mm)			
Mean ± SD	3.51 ± 2.69	2,80 ± 1.91	*P* = 0.409
Ulceration			
No	7 (30.4%)	14 (46.6%)	*P* = 0.458
Yes	7 (30.4%)	8 (26.7%)	
Unknown	9 (39.2%)	8 (26.7%)	
Treatment			
Nivolumab	21 (91.3%)	28 (93.3%)	*P* = 0.657
Nivo + Ipilimumab	2 (8.7%)	2 (6.7%)	
Previous treatment			
None (1st line)	12 (52.2%)	14 (46.7%)	*P* = 0.230
Chemotherapy	1 (4.3%)	1 (3.3%)	
Immunotherapy	0 (0%)	5 (16.7%)	
Targeted therapy	10 (43.5%)	10 (33.3%)	
Number of metastatic sites			
Mean ± SD	4,7 ± 2,0	4,7 ± 4,1	*P* = 0.186
Baseline LDH (UI/L)			
Mean ± SD	321 ± 304	180 ± 61	*P* = 0.070
Response group			
OR+	6 (26.1%)	13 (43.3%)	*P* = 0.337
OR-PD+	11 (47.8%)	9 (30.0%)	
OR-PD−	6 (26.1%)	8 (26.7%)	

The average ctDNA concentration was higher in OR-PD+ patients (581 mutated copies/mL) than in OR+ patients (91 mutated copies/mL). However, this difference was not statistically significant (*P* = 0.330).

**Figure 1:**
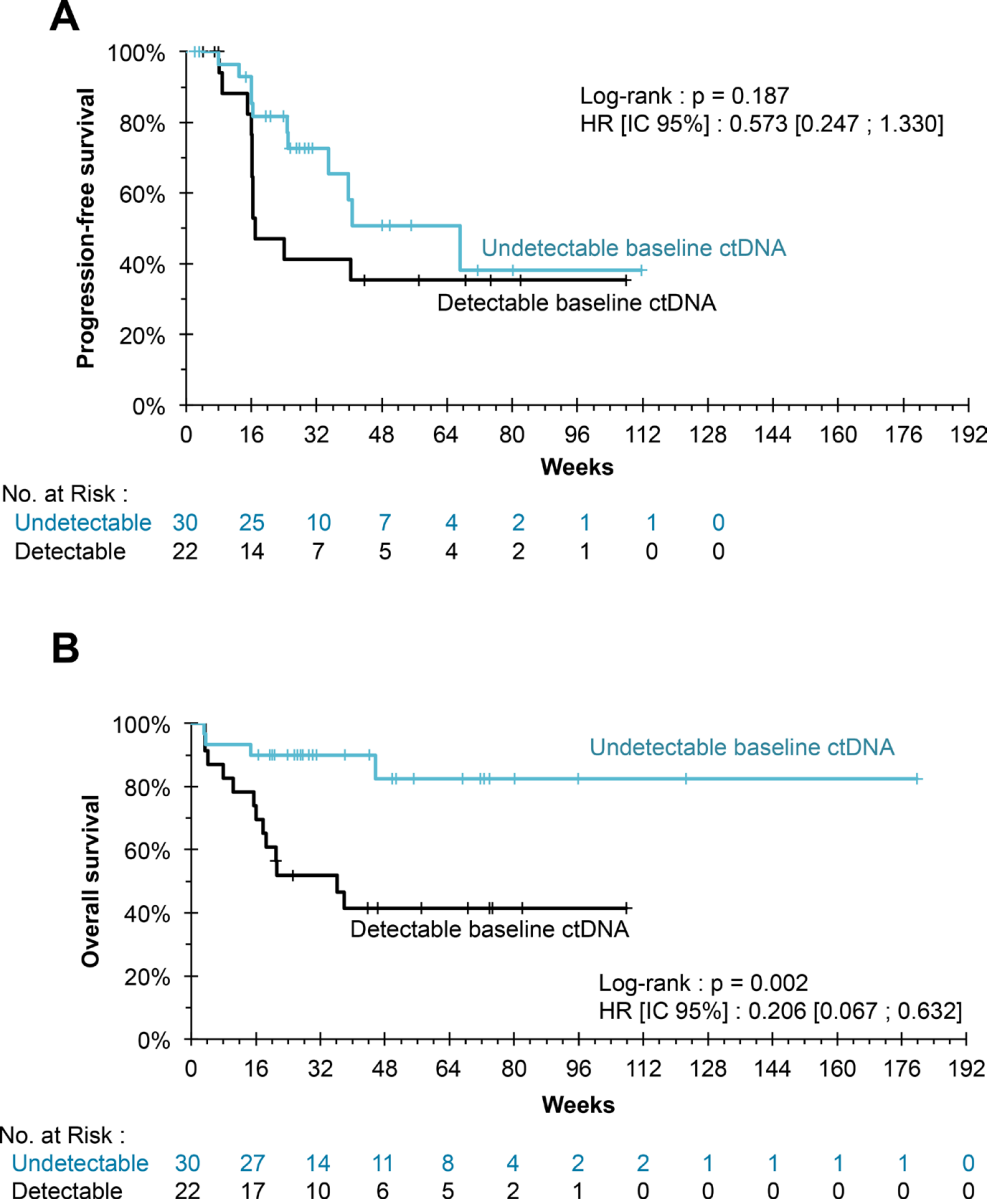
(**A**) Kaplan–Meier estimation for progression-free survival. The progression-free survival of patients whose ctDNA was undetectable at the initiation of treatment did not differ significantly from that of patients whose ctDNA was detectable (*P* = 0.187). (**B**) Kaplan Meier estimate for overall survival. The undetectability of ctDNA at treatment initiation was associated with a significant benefit in OS (*P* = 0.002).

The undetectability of ctDNA at baseline was associated with a higher 6-month survival rate (90.0%, compared to 56.2% in the case of detectable ctDNA; *P* = 0.002). However, neither ctDNA detectability nor baseline ctDNA concentration correlated significantly with PFS (*P* = 0.187 and *P* = 0.074, respectively) (Figure 1).

Longitudinal samples were analyzed for 22 patients (plasma samples were not available for one patient) whose ctDNA was detectable at baseline: 6 OR+ patients, 10 OR–PD+ patients and 6 OR-PD− patients (4 patients who died and 2 patients who showed a grade 4 adverse event before the first assessment).

### Evaluation criteria

Evaluation criteria were developed to characterize the longitudinal variations of ctDNA. The biological response (bR) was defined as a significant decrease in ctDNA compared to the baseline level, whereas the biological progression (bP) was defined as a significant increase in ctDNA compared to the nadir.

**Figure 2:**
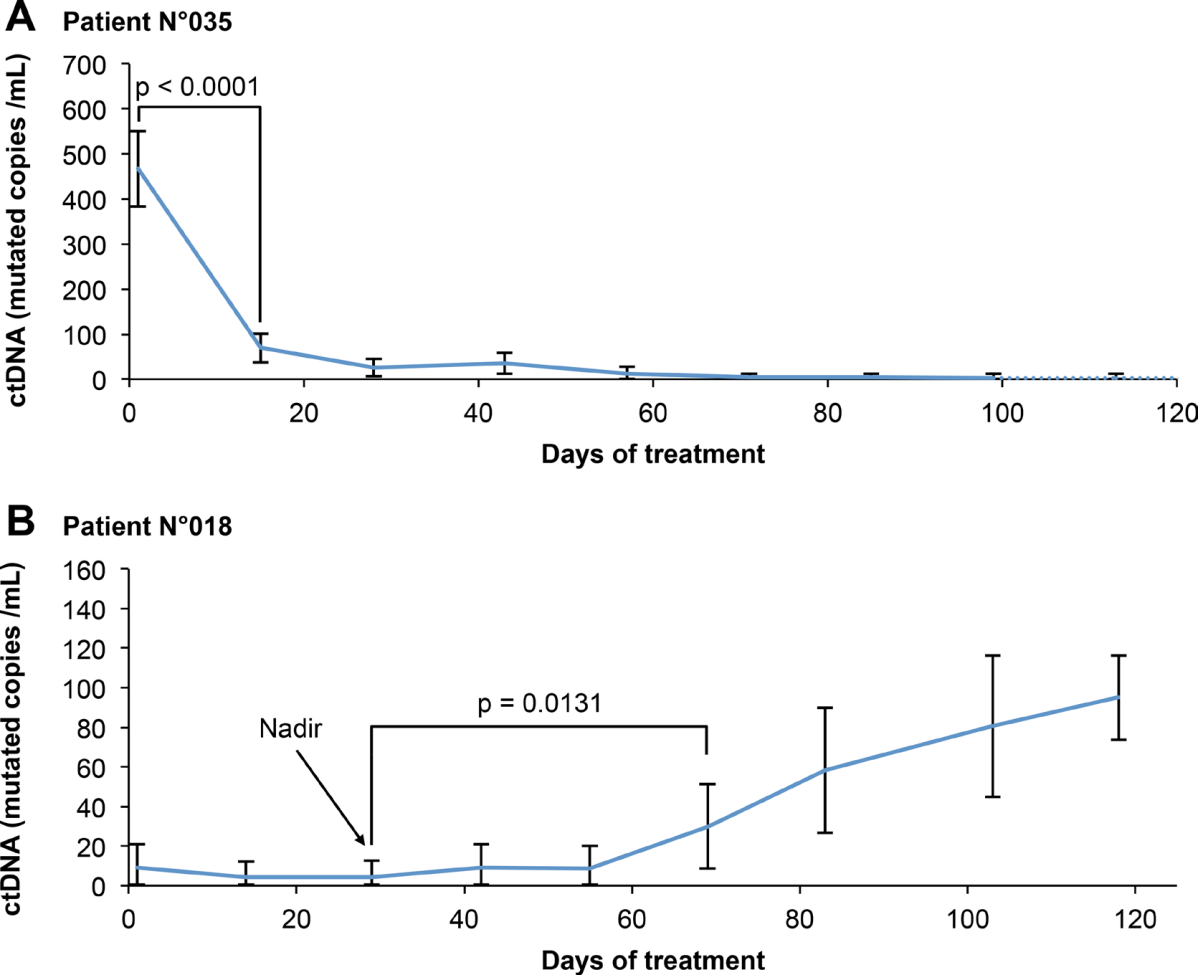
Example of use of our evaluation criteria for two representative patients (**A**) Patient of the OR + group: therapeutic response is associated with a decrease in ctDNA concentration. bR is evaluated according to the degree of significance of the decrease in ctDNA relative to the baseline, given the variability of the measure. (**B**) Patient of the OR-PD+ group: disease progression is associated with an increase in ctDNA concentration. bP is evaluated according to the degree of significance of the increase in ctDNA relative to the nadir, given the variability of the measure. Error bars represent the 95% confidence interval of each measure, calculated from the dPCR data as detailed in the method section.

A variation in ctDNA was considered if it was significantly greater than the variability of the dPCR measurement. However, the dPCR quantification precision is dependent on the concentration of the analyzed DNA extract. Therefore, it seemed inappropriate to define bR and bP using fixed thresholds. On the other hand, dPCR allows the quantification of the measurement variability. This allowed us, for each follow-up point, to compare the proportion of dPCR mutation-positive wells between this point and the baseline and nadir measurements using a statistical test (one-sided *Z*-test) (Figure 2).

**Figure 3:**
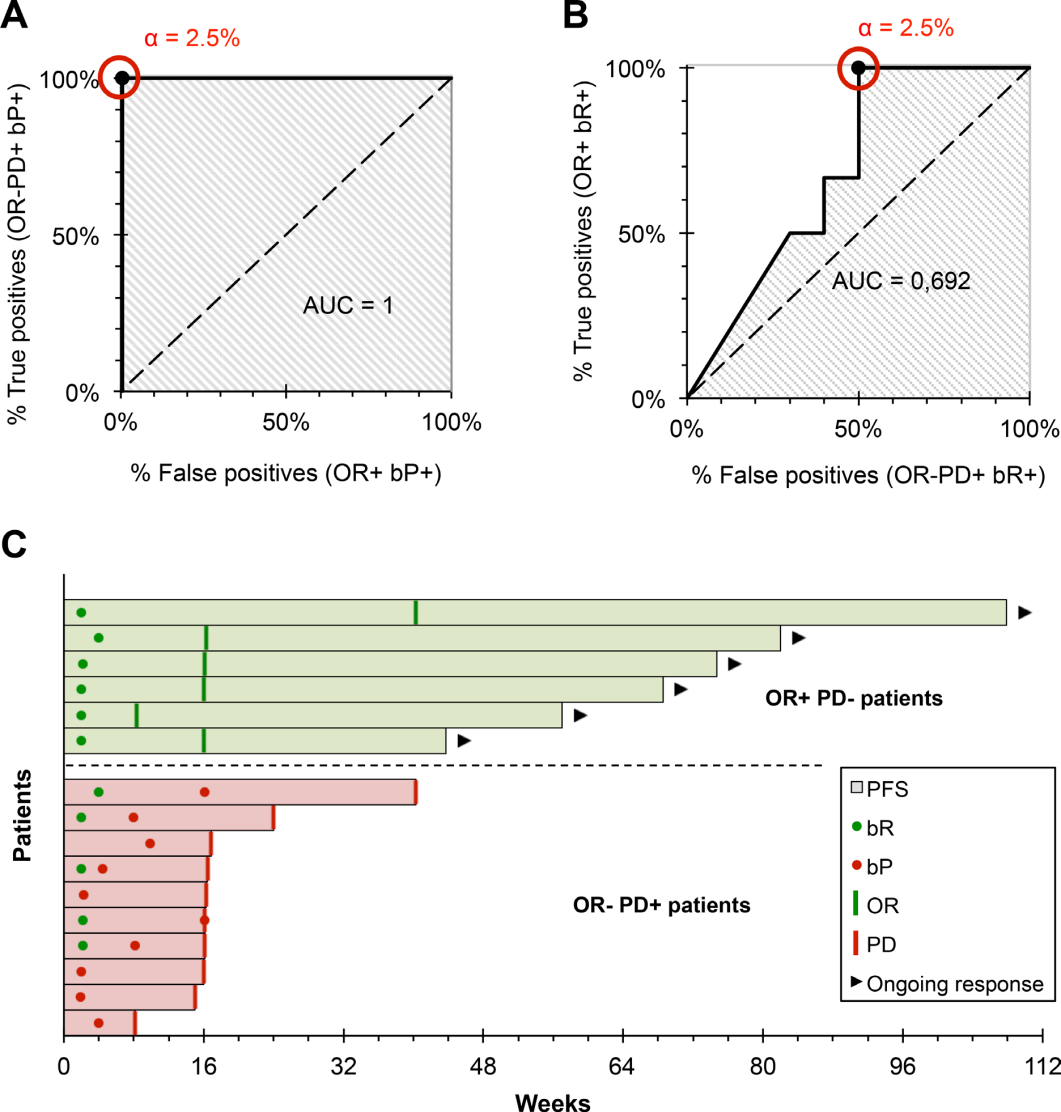
Development and evaluation of our interpretation criteria (**A**) ROC curve of the bP, for the prediction of progressive disease. For an α threshold = 2.5%, bP detected progressive disease with a sensitivity and specificity = 100% (*n* = 16). (**B**) ROC curve of the bR, for the prediction of the objective response. For an α threshold = 2.5%, bR detected progressive disease with sensitivity = 100% and specificity = 50% (*n* = 16). (**C**) Earliness of biological monitoring: bR is detected on average 115 days before the response (range: 44–268 days); bP is detected on average 79 days before the response (range: 0–169 days).

The α threshold was established using ROC curves (Figure 3A and 3B). For a threshold α = 2.5%, a bP was detected during follow-up in all OR-PD+ patients, and in no OR+ patients (i.e. sensitivity = 100% and specificity = 100% for the identification of clinical progression). bP was detected 0 to 169 days before the characterization of radiological progression in OR-PD+ patients (mean = 79 days, Figure 3C). For the same threshold, a bR was detected during follow-up in all OR+ patients (i.e. sensitivity = 100% for the identification of the clinical response) 44 to 268 days before characterization of the radiological objective response (mean = 115 days, Figure 3C). On the other hand, a bR was detectable in some OR-PD+ patients, regardless of the threshold value: the specificity of the bR for the identification of the clinical response was 50% for a threshold α = 2.5%. We were unable to demonstrate any clinical benefit associated with the detection of bR in these patients: the PFS of OR-PD+ patients did not differ significantly whether they exhibited a bR during follow-up or not (*p* = 0.105).

### Robustness

Since bR and bP were dependent on the observation of ctDNA variations during follow-up, it was necessary to evaluate the impact of a possible lack of blood samples during monitoring. We reassessed these evaluation criteria on 7700 resamples, obtained by random removal of a random number of post-baseline samples.

bR was detected with a constant sensitivity of 100% and median specificity of 50% (observed 95% CI: [33.3%; 100%]), 96.3 days prior to the radiological objective response, on the median. In OR+PD- patients, every single monitoring point met the bR criteria, which explains the constant 100% sensitivity of bR despite resampling,

bP was detected with a median sensitivity of 80% (observed 95% CI: [20%; 100%]), and a constant specificity of 100%, 65.8 days before radiological progression, on the median. By simulation, we determined that only 4 follow-up points, at weeks 2, 4, 8 and 16, were sufficient to detect bP with 100% sensitivity in OR-PD+ patients in our cohort.

### Biological follow-up

Once the interpretation criteria had been fixed, we evaluated the association between the biological monitoring and PFS and OS, for the 22 patients (including OR-PD− patients).

The absence of bR at the first assessment was associated with a 0% response rate (0/10 patients) and a 0% PFS rate at 120 days (median PFS = 112 days). The median OS was 130 days.

Likewise, in the case of initial bR, a subsequent bP at week 4, 8 or 16 was associated with a 0% response rate (0/6 patients), and median PFS and OS values of 115 and 148 days, respectively.

**Figure 4:**
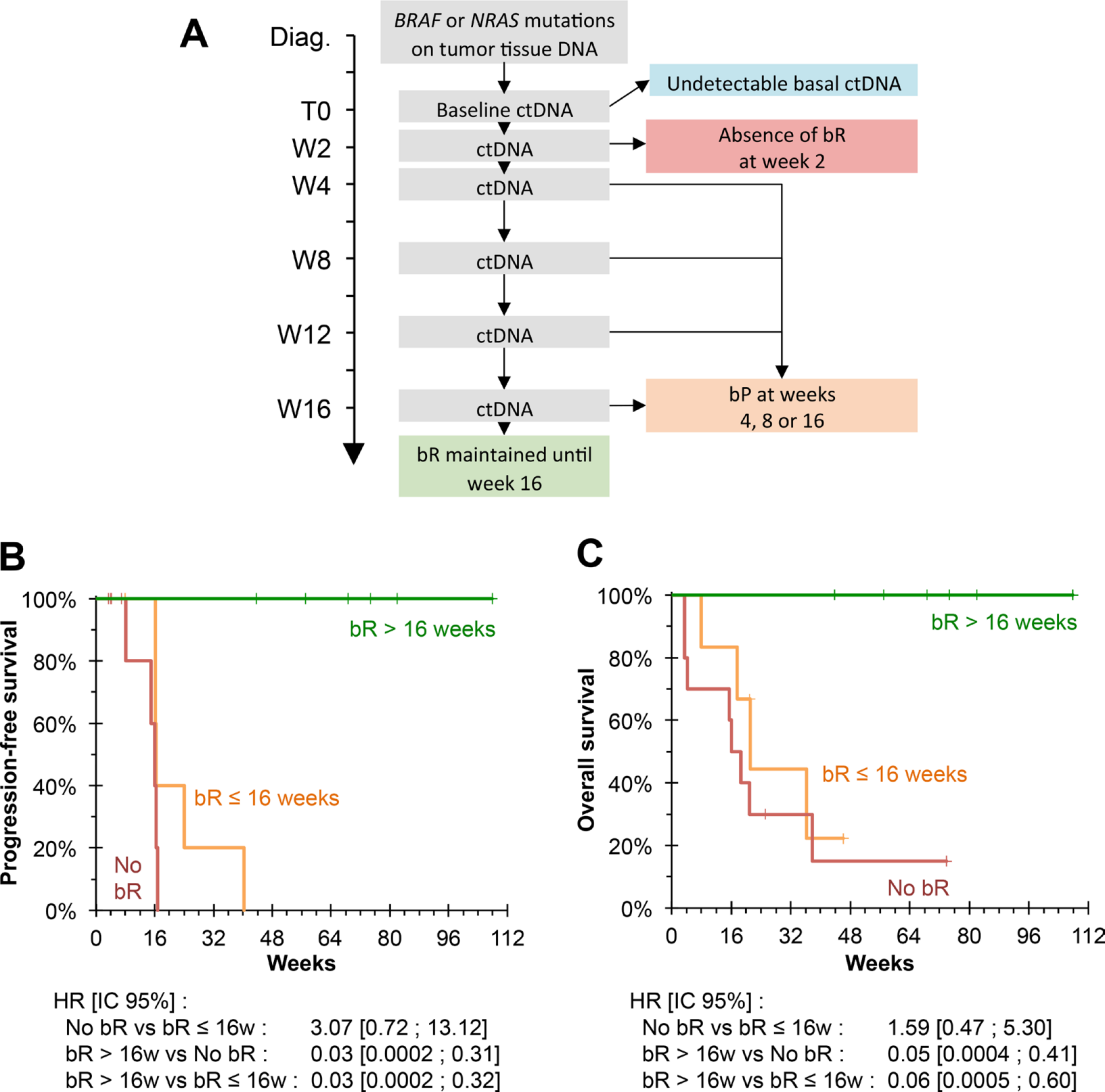
Biological follow-up (**A**) Biological monitoring model. 30 patients had an undetectable ctDNA baseline; 10 patients did not present bR at the first biological evaluation; 6 patients presented initial bR followed by bP at weeks 4, 8 or 16; 6 patients maintained a bR until week 16. (**B**) Kaplan–Meier estimate for PFS, based on biological monitoring. No patient who maintained bR until the 16th week showed any progression. PFS was significantly higher than patients who did not have bR or who did not maintain bR until week 16 (*P* = 0.001 and *P* = 0.001, respectively). (**C**) Kaplan Meier estimate for OS, based on biological monitoring. No patient who maintained bR until week 16 died. OS was significantly higher than patients who did not have bR or did not maintain bR until week 16 (*P* = 0.002 and *P* = 0.010 respectively).

Conversely, the persistence of bR up to week 16 of treatment was associated with a 100% response rate (6/6 patients) and PFS and OS rates of 100% at the time of analysis, for follow-up periods of 305 to 755 days (Figure 4).

Multivariable survival analysis showed that a sustained bR was associated with better PFS and OS independently of the parameters investigated (presence of lung metastases, presence of non-lung visceral metastases, baseline LDH activity, baseline concentration of ctDNA) after adjustment and accounting for guarantee-time bias (Table 3).

Table 3Multivariate survival analysis of progression-free survival (A) and overall survival (B) with Cox model with time-dependent covariates

**Table:** (A)

Variable	Multivariate *p*-value	HR	95% IC
bR > 16 weeks	0.003	0.012	0.00001–0.284
Presence of lung metastases	0.273	2.164	0.536–9.517
Presence of non-lung visceral metastases	0.431	0.462	0.066–3.440
Baseline LDH level	0.708	1.001	0.996–1.007
Baseline ctDNA level	0.164	0.999	0.998–1.000

**Table:** (B)

Variable	Multivariate *p*-value	HR	95% IC
bR > 16 weeks	0.014	0.067	0.0005–0.631
Presence of lung metastases	0.236	2.118	0.621–8.662
Presence of non-lung visceral metastases	0.295	2.283	0.494–12.460
Baseline LDH level	0.297	0.998	0.993–1.002
Baseline ctDNA level	0.039	1.0001	1.0000–1.0003

## DISCUSSION

Several studies have demonstrated the existence of a correlation between ctDNA kinetics and melanoma response to BRAF inhibitors [16, 18] and to anti-PD1 antibodies. However, the standardization of criteria for interpreting these variations in ctDNA over time remains a prerequisite for using ctDNA as a robust biomarker.

Our study is the first to establish quantitative biological response and biological progression criteria for the interpretation of ctDNA kinetics. We defined biological response (bR) as a significant decrease in plasma ctDNA compared to the baseline level considering the dPCR precision. Similarly, biological progression (bP) was defined as a significant increase in ctDNA compared to the nadir. Within our cohort, maintenance of a bR until week 16 was 100% predictive of a prolonged response and survival, independently of other prognostic factors. Conversely, the absence of bR as early as week 2 was associated with a 0% response rate and a short PFS (<120 days).

Qualitative evaluation criteria, based on ctDNA detectability during metastatic melanoma follow-up, have already been proposed by Lee *et al.* [17]. The application of these criteria to our cohort reproduced the results: patients whose ctDNA was initially undetectable or became undetectable before week 12 had response rates of 43% and 83% respectively (13/30 patients and 5/6 patients; compared with 72% and 77% according to Lee *et al.*), whereas the persistence of detectable ctDNA up to week 12 was associated with a response rate of 6% (1/16 patient; compared with 6% according to Lee *et al.*). OR-PD+ patients were identified on average only 43 days before radiological progression. In total, quantitative ctDNA monitoring identified responses and resistances to anti-PD1 in a more discriminating and earlier manner than qualitative criteria.

The first challenge of quantitative monitoring is that it requires an absolute quantification of ctDNA. Indeed, the quantification of the relative abundance of ctDNA to the total amount of circulating DNA may be influenced by multiple physiopathological or preanalytical factors inducing an increase in non-tumor DNA release in plasma, including inflammation, stimulation of antitumor immunity, parenchymal lysis during tumor progression or *in vitro* leukocyte lysis. This constraint requires the use of techniques allowing the absolute quantification of mutated copies, such as dPCR, or the adaptation of relative quantification techniques such as qPCR, or NGS using an internal quantification standard.

The second challenge associated with quantitative monitoring is the need to master the measurement precision. Indeed, it is essential to be able to distinguish variations related to the progression of the tumor from variations related to measurement imprecision, to interpret the variations of ctDNA over time. However, the quantification precision is highly dependent on ctDNA concentration, meaning that it is incorrect to define criteria based on relative variation thresholds (for example, an increase in ctDNA of 100% cannot be interpreted in the same way for ctDNA changes from 1 to 2 mutated copies/mL of plasma or from 1,000 to 2,000 mutated copies/mL of plasma). This constraint could explain the lack of standardization of quantitative criteria for other types of tumors, such as NSCLC [19–21], colorectal cancer [22] or breast cancer [23].

The monitoring of ctDNA required that the mutation be detectable in circulating DNA at baseline. In our cohort, the low detection rate at the initiation of treatment (43%) is one of the main limitations of our biological monitoring model. However, patients with undetectable ctDNA at baseline have a higher survival rate, and a radiological evaluation might be sufficient.

During revision of our manuscript, Lee *et al.* [24] clearly demonstrated that ctDNA monitoring can accurately differentiate pseudoprogression from true progression of disease in patients with melanoma treated with PD-1 antibodies. Unfortunately, we have not identify any patient with pseudoprogression in our cohort.

The role of quantitative monitoring of ctDNA in the therapeutic strategy remains to be determined. The interest of early detection of resistance to anti-PD1 is partly conditioned by the existence of effective therapeutic alternatives. Our biological monitoring model needs to be evaluated further in comparative clinical trials in which the treatment will be changed in the absence of early bR, or/and at bP. A similar approach is being evaluated in the APPLE clinical trial. In arm B, EGFR-mutant non-small-cell lung cancer patients will be switched from gefitinib to osimertinib treatment as soon as the T790M resistance EGFR mutation is detected in ctDNA [25].

## MATERIALS AND METHODS

### Patients and treatment

Patients who started anti-PD1 immunotherapy treatment between January 2014 and March 2017 for stage IV or unresectable stage III metastatic cutaneous melanoma with a *BRAF* or *NRAS* mutation were included in this study. All patients were treated with either nivolumab monotherapy, 3 mg/kg every 2 weeks, or a nivolumab-ipilimumab combination at 1 mg/kg and 3 mg/kg, respectively, every 3 weeks.

### Response assessment

Clinical follow-up was performed at each visit prior to treatment administration. Radiological monitoring was performed by means of a chest, abdomen and pelvis scan and a brain CT scan every 8 weeks. The tumor response to anti-PD1 was measured according to the RECIST v1.1 criteria, and evaluated by the investigators, accounting for the particularities of response to immunotherapy.

### Patient characteristics

The clinical characteristics of the patients were provided by the Melanoma Research and Clinical Investigation network (RIC-Mel network). These data included age, gender, stage of disease, primary tumor thickness and ulceration, number and location of metastases, baseline LDH activity, and number and nature of previous therapeutic lines.

### Samples

Plasma samples were collected for each patient before treatment initiation, after 2 weeks and 4 weeks of treatment, then every 4 weeks until progression, death or unacceptable toxicity. The samples were collected in EDTA tubes (Greiner Bio-One), centrifuged at 2000 g for 10 min, decanted and frozen at −80° C within 4 hours after collection.

### Circulating DNA analysis

ctDNA was extracted from 2 mL of plasma, thawed and re-centrifuged, using the QIAamp Circulating Nucleic Acid Kit (QIAGEN), and eluted in 50 μL of elution buffer as recommended by the supplier. DNA extracts were frozen at −20° C until analysis.

We then quantified the ctDNA for each patient, using a digital PCR (dPCR) specific for the mutations detected in the tumor tissues. The QuantStudio 3D Digital PCR System (LifeTechnologies) was used. For each sample, a reaction mixture of 15 μL was prepared with 6.5 μL of DNA extract, 7.5 μL of a PCR mix comprising Taq polymerase, dNTPs and ROX reference dye, and 1 μL of a solution containing the primers adapted to the genomic region of interest and two Taqman probes: one specific for the mutation, labeled with the FAM fluorophore, and the other, specific for the wild-type allele, labeled with the VIC fluorophore (Supplementary Table 1). All these assays were tested against a genomic DNA negative control and were found to be highly specific (no positive well). A sample was thus considered positive when containing at least 2 mutated copies per assay, i.e. 8 mutated copies/mL of plasma in our conditions of extraction and analysis.

This mixture was then partitioned onto a 20,000 well-chip by diffusion, using a semi-automatic device to standardize this step.

After sealing the chips, the amplification reaction was carried out using a suitable thermal cycler, according to the following program: hold 10 min at 96° C and then 39 cycles alternating for 2 min at 60° C and 30 s at 98° C. At the end of the amplification reaction, the fluorescence emitted by each well was read using a dedicated reader: a green FAM fluorescence signal at 518 nm was emitted by the well in the presence of the mutation, while a yellow VIC signal at 554 nm was emitted by the well in the presence of the wild-type allele. These fluorescence data were then analyzed using a software of our design (unpublished) which provides the proportion of mutation-positive wells.

This proportion of mutation-positive wells is an estimator of the probability that a well contains mutated copies. Given the number of wells filled with PCR reaction mix (ROX positive), it is possible to calculate the number of mutated copies of the assay and its 95% confidence interval, using the Poisson's law.

The measurement variability was calculated from this confidence interval, and the number of mutated copies per mL of plasma has been deduced, considering the parameters of ctDNA extraction and analysis.

### Statistical analysis

The Kruskall–Wallis test and the Chi-square test were used to compare patient characteristics. The criteria we established for the evaluation of ctDNA kinetics were defined and evaluated using ROC curves. The survival probabilities were estimated using the Kaplan–Meier method and compared using a log-rank test. Finally, the association between biological response criteria and OS or PFS were estimated using a Cox proportional hazards model with time-dependent covariate and evaluated using the Wald test. The Firth's penalized likelihood was used, because of the monotone likelihood associated with some variables. The variables included in the multivariate analysis were the presence of lung metastases, the presence of non-lung visceral metastases, baseline LDH activity, and baseline concentration of ctDNA.

All tests were conducted using bilateral assumptions and a significance level *p* < 0.05 was used to establish the significance of our observations. Statistical analyses of this study were performed using the XLSTAT and R software programs.

### Ethics statement

This study was authorized by the Nantes Ethics Group for matters pertaining to Health (GNEDS) as well as by the French National Data Protection Commission (CNIL). All patients signed a written consent form, authorizing blood sampling, storage of the samples in a bio-collection and their use for research purposes.

## SUPPLEMENTARY MATERIALS AND TABLES



## Abbreviations

BP: biological progression
BR: biological response
ctDNA: circulating tumor DNA
dPCR: digital PCR
OS: overall survival
PFS: progression free survival
