# Supplementary material for: Quantitative monitoring of circulating tumor DNA predicts response of cutaneous metastatic melanoma to anti-PD1 immunotherapy
Source: Oncotarget. 2018 May 18;9(38):25265–76. doi: 10.18632/oncotarget.25404 (PMC5982743; doi:10.18632/oncotarget.25404)
Supplement: Supplementary file 1 [file oncotarget-09-25265-s001.pdf]

Quantitative monitoring of circulating tumor DNA predicts response of cutaneous metastatic melanoma to anti-PD1 immunotherapy

SUPPLEMENTARY MATERIALS

Supplementary Table 1: References of the oligonucleotide probes used for dPCR analysis

Gene	Mutation		Reference*	Patients (*n*)	Wild type allele (VIC)	Mutant allele (FAM)
BRAF	p.V600E	c.1799T>A	AH6R5PH	21	A	T
	p.V600K	c.1798_1799GT>AA	AHBKFRM	3	AC	TT
NRAS	p.G12D	c.35G>A	AHI15D6	1	C	T
	p.Q61K	c.181C>A	AH1SDFJ	10	G	T
	p.Q61L	c.182A>T	AHD2CUE	1	T	A
	p.Q61R	c.182A>G	AHS1P6Q	17	T	C

*Applied Biosystems Thermo Fisher Scientific.
